# Disparities in the PrEP continuum for trans women compared to MSM in San Francisco, California: results from population‐based cross‐sectional behavioural surveillance studies

**DOI:** 10.1002/jia2.25539

**Published:** 2020-06-30

**Authors:** Erin C Wilson, Caitlin M Turner, Sean Arayasirikul, Marguerita Lightfoot, Susan Scheer, Henry F Raymond, Albert Liu

**Affiliations:** ^1^ Center for Public Health Research San Francisco Department of Public Health San Francisco CA USA; ^2^ Division of Prevention Science Center for AIDS Prevention Studies University of California San Francisco CA USA; ^3^ HIV Epidemiology San Francisco Department of Public Health San Francisco CA USA; ^4^ Rutgers University New Jersey USA; ^5^ Bridge HIV San Francisco Department of Public Health San Francisco CA USA

**Keywords:** transgender women, HIV, pre‐exposure prophylaxis, PrEP, disparities

## Abstract

**Introduction:**

Although transgender women (trans women) often are conflated with men who have sex with men  (MSM) in HIV research and services, there are distinct population differences that are important for implementing effective HIV prevention. Our objective was to examine pre‐exposure prophylaxis (PrEP) disparities between the two populations and compare individual, social and structural factors that influence differences between MSM and trans women along the PrEP continuum.

**Methods:**

We analysed data from two population‐based studies, one with trans women (Trans*National Study, 2016 ‐ 18) and the other with MSM (National HIV Behavioral Surveillance, 2017). Trans women were recruited via respondent‐driven sampling and MSM using time location sampling. Key indicators of the PrEP continuum were evaluated, including awareness, health insurance, provider discussions, recent use and adherence. Associations were also examined for PrEP continuum indicators and structural barriers (e.g. employment, homelessness).

**Results:**

Transwomen were more likely than MSM to be Latino/a (30.4% vs. 25.8%; prevalence ratio (PR)=1.08, 95% CI 1.02 to 1.14) or African American (7.1% vs. 4.5%; PR = 1.12, 1.02 to 1.24), live at or below the poverty limit (70.7% vs. 15.8%; PR = 1.47; 1.41 to 1.53), be unemployed (50.1% vs. 26.3%; PR = 1.18, 1.13 to 1.24), be homeless (8.4% vs. 3.5%; PR = 1.15, 1.06 to 1.25) and to have less than a college degree (PR = 1.41, 1.34 to 1.48). Trans women were more likely than MSM to have health insurance (95.7% vs. 89.7%, PR = 1.17, 1.06 to 1.28), but less likely than MSM to have heard of PrEP (79.1% vs. 96.7%; PR = 0.77, 0.73 to 0.81), talked with a provider about PrEP (35.5% vs. 54.9%; PR = 0.87, 0.83 to 0.91) and less likely than MSM to have used PrEP in the past six months (14.6% vs. 39.8%; PR = 0.80, 0.76 to 0.84). Among PrEP users, trans women were less likely to report being adherent to PrEP than MSM (70.4% vs. 87.4%; PR = 0.80, 0.70 to 0.91).

**Conclusions:**

We found PrEP disparities for trans women compared to MSM and the need for differentiated implementation strategies to meet the specific PrEP barriers trans women face. Inclusion of trans women’s HIV risks is needed in CDC guidance for PrEP. Interventions to increase trans women’s awareness of PrEP including at the provider and community level are also needed. Finally, programming that addresses trans women’s barriers to housing and income is also needed to reduce PrEP disparities.

## 1 INTRODUCTION

In San Francisco, men who have sex with men (MSM) and transgender women (trans women) comprised 84% of new HIV diagnoses in 2015. There has been a significant decline in new HIV infections among MSM, but new infections among trans women remain persistently high [1, 2]. Data from population‐based behavioural surveillance studies found that almost half (39%) of trans women may be living with HIV in San Francisco compared to a quarter of cisgender MSM (26.3%) [3, 4]. Meanwhile, pre‐exposure prophylaxis (PrEP) awareness and use are increasing among MSM in the United States (US) [5, 6] and in San Francisco [7]. Yet data from San Francisco showed that only 14% of 233 trans women were aware of PrEP one year after FDA approval, and little data points to improvements.

Although trans women often are conflated with MSM in HIV research and services [8], there are distinct differences between these two populations that are important for effective HIV prevention. For example, trans women face a number of unique barriers that can impact PrEP engagement, including concerns about side effects and the  effect of PrEP and gender‐affirming hormones [9], substance use and mental health issues [10, 11], incarceration [12], lack of social support [10], trauma/violence [11, 13], family rejection [14], HIV stigma [15],  anti‐trans stigma [16, 17], economic vulnerability [18] and housing instability [19, 20]. Distrust of medical institutions and lack of access to trans‐friendly providers are also significant barriers to engaging trans people in prevention services [21]. San Francisco is unique in that it has numerous trans‐specific health clinics and providers; yet trans women still face barriers to health care, including prior anti‐trans discriminatory experiences in healthcare settings, limitations in protocols to meet their healthcare needs and difficulties with sexual and drug use disclosure tied to their immigration status and/or sex work engagement [22, 23, 24]. Also, some trans women may be concerned about the impact of PrEP on hormone therapy or be worried that hormones reduces the efficacy of PrEP [25].

Data‐driven approaches to HIV prevention can identify gaps in the PrEP continuum and highlight intervention targets. We compared the PrEP continuum between MSM and trans women in San Francisco to determine if there are disparities in PrEP awareness, provider discussions, use and adherence. We also explored the individual, social and structural factors that influenced differences between MSM and trans women along the PrEP continuum using two population‐based studies. Both studies were conducted in San Francisco in overlapping periods of time with the similar recruitment and PrEP indicators. Poisson binomial regression was used to compare prevalence of PrEP continuum outcomes between populations in general and based on PrEP eligibility per CDC guidelines. Findings from this analysis will inform data‐driven efforts to address disparities in PrEP and differentiate PrEP delivery for trans women in San Francisco.

## 2 METHODS

We conducted a secondary analysis of data from population‐based studies of HIV‐uninfected trans women enrolled in the Trans*National Study (June 2016 to March 2018) and HIV‐uninfected MSM enrolled in the local National HIV Behavioral Surveillance (NHBS) MSM cycle (August to December 2017). Study recruitment and enrolment methods for both studies are described elsewhere [18, 26]. Briefly, Trans*National is a population‐based cohort study of HIV incidence among trans women in the San Francisco Bay Area. Trans women were recruited using respondent‐driven sampling. Participants age 18 years old and older who identified as a gender other than man who were male sex assigned at birth were eligible to participate. NHBS was a population‐based, cross‐sectional study of MSM in San Francisco recruited using time location sampling. Participants who were age 18 years or older and ever had sex with another man were eligible to participate. Data for NHBS were collected between August and December of 2017. Both studies included English and Spanish‐speaking participants. All participants provided written informed consent for the survey and HIV testing. Ethical approval for human subjects was obtained by the Human Research Protection Program (HRPP) at the University of California San Francisco.

### 2.1 Measures

Baseline participant demographics were assessed and compared for Trans*National and NHBS participants. Age was a continuous variable, defined as participants’ year of age at the time of taking the baseline survey. Race/ethnicity was an indicator variable of participants’ self‐reported racial/ethnic identities. Categories were defined according to Office of Management and Budget standards [27] and further collapsed into the following categories: Black or African American (non‐Hispanic or Latino/a), Latino/a or Hispanic, Other (non‐Hispanic or Latino/a Asian, American Indian/Alaska Native, Native Hawaiian or other Pacific Islander, Other or multiracial) and White (non‐Hispanic or Latino/a). Education level was categorized as possession of a high school degree, general education diploma or GED (i.e. a high school equivalency diploma for people who did not finish secondary education) or less versus having some college versus having a college degree or more. Participants provided their annual income. We re‐coded income as below the poverty line (US $25,000), at or above the poverty line, or unknown based on the extremely low‐income limit for affordable housing programmes in San Francisco [28]. Employment was dichotomized as either employed or unemployed. We also described and compared the prevalence of homelessness (i.e. living on the street or in a shelter, including living in a single room occupancy for the NHBS MSM cycle) and history of incarceration. We also examined healthcare access among participants in Trans*National and NHBS. We assessed whether participants saw a healthcare provider in the last 12 months, whether they currently had health insurance, and the type of insurance they possessed (public, private or a combination of public and private insurances). Sexual behaviours were also compared for trans women and MSM. Specifically, we examined number of condomless anal intercourse partners in the last six months, and the percent of participants’ HIV‐uninfected sexual partners who were on PrEP.

PrEP awareness was measured by asking participants, “Have you heard of PrEP before today?” Having talked to a provider about PrEP was asked as, “Have you discussed PrEP with your primary healthcare provider in the last 12 months?” PrEP use was considered having taken PrEP within the last six months. PrEP adherence was measured differently in each study. For Trans*National, we asked, “In the last seven days, how many days did you miss a dose of PrEP?,” and in NHBS we used, “In the last 30 days, have you taken PrEP every day, almost every day, or less often?” Being adherent to PrEP was defined as having taken PrEP at least four times in the past week (Trans*National) or every day or almost every day (NHBS), a level of pill‐taking associated with high levels of protection from HIV in prior studies [29, 30]. In Trans*National, PrEP awareness and ever having used PrEP was assessed at baseline, whereas data on having ever talked to a provider about PrEP and PrEP adherence were assessed at the six‐month follow‐up assessment; for NHBS, these questions were asked at the one‐time survey visit. PrEP candidacy also measured based on CDC guidelines for MSM because there are no trans‐specific guidelines [31].

### 2.2 Data analysis

The present analysis was restricted to trans women and MSM who were not living with HIV. Our Trans*National data set is a combination of socio‐demographics and structural factors assessed at baseline, and PrEP indicators assessed at baseline and six months. Only trans women not living with HIV who completed every item measured for this analysis in their baseline and six‐month follow‐up assessment were included. Out of 428 HIV‐negative participants at baseline, we retained 369 (86%) trans women who had completed their six‐month assessments. Of the 497 MSM in NHBS who provided self‐report of their HIV status, 399 MSM were included once we restricted to HIV‐negative participants. We concatenated the MSM and trans women data sets, and adjusted for MSM versus trans women as an exposure variable in the comparison of outcomes.

First, we characterized the study samples by comparing socio‐demographics and health care access among trans women and MSM using prevalence ratios (PRs) estimated from bivariable Poisson binomial regression models. Then, key steps of the PrEP continuum were evaluated using all data available within the restricted Trans*National (n = 369) and NHBS (n = 399) databases. These steps included PrEP awareness, discussing PrEP with a provider, PrEP use in the past six months and taking PrEP daily/almost daily (NHBS) or ≥4 times in the past week (Trans*National). Differences in PrEP continuum steps for trans women compared to MSM were also estimated with bivariable Poisson binomial regression models adjusting for race/ethnicity and homelessness, given the a priori differences hypothesized in study selection and PrEP outcomes by these factors for trans women compared to MSM. Controlling for race/ethnicity and homelessness was done to allow for an unbiased comparison between these two groups (i.e. MSM and trans women) given the differences in race/ethnicity diversity and homelessness and the importance of these factors on risk, especially for trans women [18, 32].

Key steps in the PrEP continuum were also calculated among MSM and trans women considered candidates for PrEP based on CDC criteria [31], including: (1) being 18 years of age or older, (2) being HIV‐negative, (3) having any male sex partners in the last six months and (4) having a non‐monogamous HIV‐negative male partner and having condomless anal intercourse or having an STD or having an HIV‐positive primary partner. Prevalence ratios from Poisson binomial regression were used to compare prevalence of PrEP continuum outcomes for PrEP candidates who were trans women versus MSM.

## 3 RESULTS

### 3.1 Differences in socio‐demographics and health care access between trans women and MSM

Trans women were significantly more likely than MSM to be Latino/a (30.4% vs. 25.8%; prevalence ratio [PR] = 1.08, 95% CI 1.01 to 1.14) or African American (7.1% vs. 4.5%; PR = 1.12, 1.02 to 1.24) than white (see Table 1). Trans women were significantly more likely than MSM to be living at or below the poverty level (70.7% vs. 15.8%; PR = 1.47; 1.41 to 1.53), unemployed (50% vs. 26%; PR = 1.18, 1.13 to 1.24) and homeless (8.4% vs. 3.5%; PR = 1.15, 1.06 to 1.25), and trans women were more likely to have ever been incarcerated than MSM (52.6% vs. 15.3%; PR = 1.31, 1.26 to 1.37). Trans women were also significantly more likely than MSM to have less than a college degree (39.6% vs. 10.5%, PR = 1.41 for a high school degree or GED, and PR = 1.27 for some college or a technical degree).

**Table 1:** Participant characteristics for MSM in NHBS and trans women in Trans*National

	MSM		Trans women		Bivariable comparison	
	N	%	N	%	PR	95% CI
Total	399	100.00	369	100.00		
Demographic						
Age (years), Median & IQR	36	29 to 49	37	27 to 51	1.00	1.00 to 1.00
Race/ethnicity						
White	205	51.4	145	39.3	Ref	
Black or African American	18	4.5	26	7.1	1.12	1.02 to 1.24*
Hispanic/Latino/a	103	25.8	112	30.4	1.08	1.02 to 1.14*
Other	72	18.1	86	23.3	1.09	1.03 to 1.16**
Education						
Some college/technical degree	83	20.8	127	34.4	1.27	1.21 to 1.35**
College degree and above	274	68.7	96	26.0	Ref	
HS/GED or less	42	10.5	146	39.6	1.41	1.34 to 1.48**
Annual income						
Above poverty limit	335	84.0	99	26.8	Ref	
At or below poverty limit	63	15.8	261	70.7	1.47	1.41 to 1.53**
Employed						
No	105	26.3	185	50.1	1.18	1.13 to 1.24**
Yes	294	73.7	184	49.9	Ref	
Currently homeless						
No	385	96.5	338	91.6	Ref	
Yes	14	3.5	31	8.4	1.15	1.06 to 1.25**
Ever incarcerated						
No	337	84.5	175	47.4	Ref	
Yes	61	15.3	194	52.6	1.31	1.26 to 1.37**
Health care						
Saw a healthcare provider in the last 12 months						
No	30	7.5	46	12.5	Ref	
Yes	369	92.5	321	87.0	0.91	0.85 to 0.98*
Currently has health insurance						
No	41	10.3	16	4.3	Ref	
Yes	358	89.7	353	95.7	1.17	1.06 to 1.28**
Insurance type						
None	41	10.3	16	4.3	Ref	
Public	87	21.8	239	64.8	1.35	1.23 to 1.49**
Private	258	64.7	100	27.1	1.00	0.91 to 1.10
Public + private	13	3.3	6	1.6	1.03	0.86 to 1.23
Sexual behaviours & health outcomes						
# condomless anal intercourse partners, last six months Median and IQR	1	0 to 2	0	0 to 0	0.88	0.86 to 0.89**
Percent of HIV‐uninfected partners on PrEP						
0	152	38.1	191	51.8	Ref	
(0, 25]	36	9.0	7	1.9	0.75	0.68 to 0.83**
(25, 50]	50	12.5	4	1.1	0.69	0.64 to 0.74**
(50, 75]	40	10.0	1	0.3	0.66	0.62 to 0.70**
(75, 100]	42	10.5	6	1.6	0.72	0.66 to 0.79**
No uninfected partner	79	19.8	160	43.4	1.07	1.02 to 1.1388

Percentages column‐calculated out of total sample (n = 399 for NHBS; n = 369 for T × N). CI, 95% confidence interval; PR, crude prevalence ratio from Poisson binomial regression comparing prevalence of PrEP continuum steps for trans women to that for MSM; Ref, reference group.

a *p* < 0.05

b *p* < 0.01MSM: men who have sex with men; HS: high school; GED: General Educational Development; PrEP: pre‐exposure prophylaxis; NHBS: National HIV Behavioral Surveillance

In terms of health care, trans women were significantly more likely than MSM to have health insurance (95.7% vs. 89.7%, PR = 1.17, 1.06 to 1.28) and to have public rather than private health insurance (64.8% vs. 21.8%, PR = 1.35, 1.23 to 1.49), but significantly less likely than MSM to have seen a healthcare provider in the last 12 months (86.9% vs. 92.5%, PR = 0.91, 0.85 to 0.98).

### 3.2 PrEP continuum among trans women compared to MSM overall

**Figure 1:**
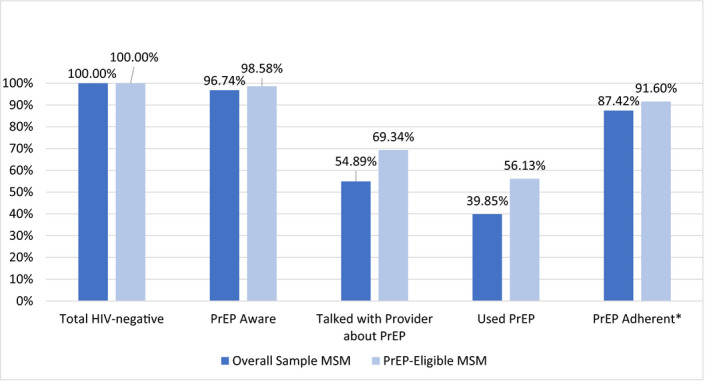
PrEP continuum indicators for MSM, overall and by PrEP‐eligibility, San Francisco, USA, 2016/2017. *Denominator: those who reported using PrEP in the last six months. MSM, men who have sex with men; PrEP, pre‐exposure prophylaxis

Trans women reported significant disparities along the PrEP continuum compared to MSM (see Figure 1). Significantly fewer trans women than MSM were aware of PrEP (292/369 = 79.1% vs. 386/399 = 96.7%, aPR = 0.83, 0.77 to 0.88, *p* < 0.01), had used PrEP within the last six months (54/369 = 14.6% vs. 159/399 = 39.9%, PR = 0.36, 0.28 to 0.47, *p* < 0.01), talked with a provider about PrEP (131/369 = 35.5% vs. 219/399 = 54.9%; PR = 0.62, 0.53 to 0.73, *p* < 0.01). Among the 54 trans women and 159 MSM PrEP users, trans women were less likely to report being adherent to PrEP (70.4% vs. 87.4%; PR = 0.82, 0.68 to 0.99, *p* = 0.04).

### 3.3 PrEP candidacy for trans women and MSM

Over one‐half of MSM in our data set would be considered PrEP candidates based on CDC criteria (212/399, 53.1%), but only 15.7% of trans women (58/369) would have been considered candidates. Based on CDC guidelines, significantly fewer trans women would be PrEP candidates due to fewer trans women than MSM reporting any male sexual partners in the last six months (157/369 = 42.5% vs. 277/369 = 69.4%, *p* < 0.01). Similarly, fewer trans women compared with MSM reported having one or more PrEP candidacy criteria (non‐monogamous sex with a HIV‐negative male partner and condomless anal intercourse; having a sexually transmitted disease; or having a primary partner who was living with HIV) (15.7% vs. 53.1%, *p* < 0.01).

### 3.4 PrEP continuums among trans women and MSM candidates based on CDC criteria

**Figure 2:**
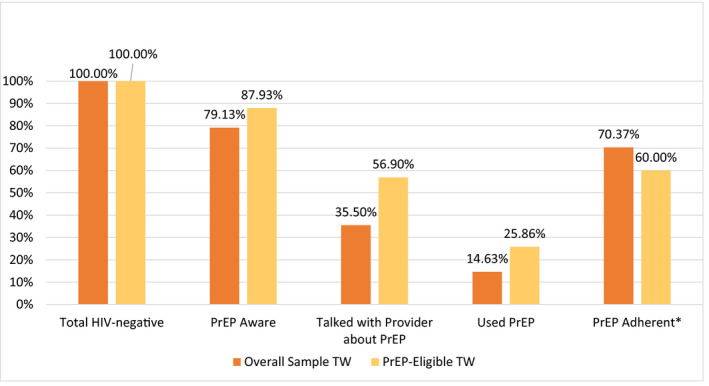
PrEP continuum indicators for trans women (TW), overall and by PrEP‐eligibility, San Francisco, USA, 2016/2017. *Denominator: those who reported using PrEP in the last six months. PrEP, pre‐exposure prophylaxis

Figure 1 depicts the PrEP continuum for MSM from the overall sample and from the PrEP‐eligible sample; Figure 2 accomplishes this for trans women from the overall sample and from the PrEP‐eligible sample. Of MSM PrEP candidates, 98.6% (209/212) were aware of PrEP, 69.3% (147/212) had talked with a provider about PrEP, 56.1% (119/212) had used PrEP in the last six months and 91.6% (109/119) of PrEP users reported being adherent to PrEP. Of trans women candidates, 87.9% (51/58) were aware of PrEP, 56.9% (33/58) had talked with their provider about PrEP, 25.9% (15/58) had used PrEP in the last six months and 60.0% (9/15) of PrEP users reported being adherent to PrEP. When comparing trans women and MSM candidates, there were significantly fewer trans women candidates aware of PrEP than MSM (87.9% vs. 98.6%, aPR = 0.90, CI = 0.81 to 0.99, *p* = 0.04), and significantly fewer trans women than MSM candidates who had used PrEP (25.9% vs. 56.1%) in the last six months (aPR = 0.50, CI = 0.31 to 0.78, *p* < 0.01) after adjusting for race/ethnicity and homelessness. There were no significant adjusted differences in the prevalence of trans women vs. MSM who had spoken to their provider about PrEP (56.9% vs. 69.3%, aPR = 0.85, CI‐.66 to 1.09, *p* = 0.19) or of being adherent to PrEP if taking it (60% vs. 91.6%, aPR = 0.79, CI = 0.58 to 1.08, *p* = 0.14).

## 4 DISCUSSION

Our data point to marked disparities in the PrEP continuum for trans women compared to MSM in San Francisco. Data on lower awareness among trans women are consistent with other local research showing that trans women did not believe PrEP was for them because PrEP social marketing campaigns initially focused exclusively on MSM in San Francisco [8]. Conversely, a PrEP campaign inclusive of trans women in Chicago, Illinois did not find differences in awareness between MSM and trans women [33]. Low reporting of trans women’s participation in efficacy trials may have also impacted the community’s awareness of PrEP [34].

Provider willingness to prescribe and healthcare avoidance due to stigma may have presented additional barriers to awareness and uptake of PrEP among trans women. Research finds that providers support the provision of PrEP as a HIV prevention public health intervention, but knowledge, acceptance and willingness to prescribe it has been limited [35]. Many providers also do not offer PrEP in the course of their clinical practice [36]. Lower education was also found among trans women in our study compared to MSM, which may have impacted trans women’s health literacy and power to engage in a discussion with providers about PrEP [37]. Trans women in our study were also less likely than MSM to have seen a health care provider in the last year, despite having higher levels of health insurance. Studies have found that trans women face considerable stigma from medical providers, which may cause healthcare avoidance [38, 39]. Discrimination from HIV care providers created reluctance of African American trans women in one study to see their doctor [40]. Healthcare avoidance may have also been precipitated by fear of disclosing trans identity, lack of cultural competence by providers or structural barriers like transportation costs [41, 42]. Trans women may also have avoided asking for PrEP because of discomfort discussing sexual and drug use behaviours with their primary care providers [37]. Provider barriers and healthcare avoidance among trans women are an important focus of efforts to better engage trans women in PrEP.

Although the two data sets are different for MSM and trans women, they each point to factors that influenced PrEP awareness, access and uptake for the respective populations that allow for comparisons. Structural barriers of having low income, unemployment, homelessness and incarceration were all significantly more prevalent among trans women than MSM. More than half of trans women in our sample had been previously incarcerated compared to 15% of MSM. High incarceration among trans women in our sample is consistent with findings from other studies of trans women [43, 44]. Having a history of incarceration may have impacted trans women’s current housing, income and employment opportunities. The elevated presence of competing priorities for survival from lack of income may have limited trans women’s awareness or interest in PrEP. Structural barriers may have also impacted adherence among those on PrEP. Trans women reported disproportionately high homelessness compared to MSM. Unstable housing was similarly a barrier to viral suppression among trans women in a recent analysis from San Francisco [32]. Not having a place to store and privately take medication may explain the lower PrEP adherence among trans women compared to MSM in our study [45].

We also found that significantly fewer trans women would be considered candidates for PrEP compared to MSM based on CDC guidelines. Research is increasingly uncovering that HIV transmission among trans women is varied and different from MSM [46]. CDC guidelines for the population given the high HIV prevalence of HIV and specific risks trans women face remain inadequate [32].

The primary limitation to this study is that it was not designed to compare the PrEP continuum in these populations. Measures, and therefore, data compared for this analysis had differences in how the survey questions were asked in Trans*National compared to NHBS. For example the adherence measure from the sample of trans women asked how many days participants took their medications in the last week. We tried to address differences in how the data were captured by creating measures as conservatively as possible. This measure was then re‐categorized to a month‐long recall window to be comparable to the MSM sample, and re‐grouped to be qualitatively comparable to the “every day or almost every day” versus “less often” language used in the survey of MSM. It is possible that trans women’s adherence fluctuated week to week, and therefore those who reported four to seven days of PrEP, but actually averaged 16 days of PrEP in the last 30 would be misclassified as PrEP adherent. Even so, this would actually over‐represent the number of trans women who were PrEP adherent, and therefore produce more conservative estimates for the hypothesized differences between trans women and MSM. Thus, the disparity in PrEP adherence that we found may actually be more severe and stronger in magnitude, but our conclusion would remain qualitatively the same (i.e. that MSM are more adherent to PrEP than trans women). For the PrEP‐eligible sample, this misclassification could have biased results toward the null and may, in addition to the smaller number of trans women in this restricted analysis, explain why we did not find statistically significant differences in PrEP adherence for PrEP‐eligible MSM compared to PrEP‐eligible trans women. Also, study data collection periods overlapped and were not entirely synced, which we could not account for in the measures or analysis. All PrEP continuum indicators were by self‐report, including PrEP use and adherence, so we do not know conclusively if PrEP was used and adhered to at levels we observed in each population. Lastly, for the comparison of PrEP use and adherence levels between MSM and trans women, there are power limitations given that only 15 trans women reported using PrEP. Despite limitations, this data‐informed approach to assessing the PrEP continuum was a useful tool for identifying PrEP disparities between trans women and MSM and helped identiyf potential points of intervention.

## 5 CONCLUSIONS

Our study points to the need for differentiated PrEP implementation strategies to meet the barriers trans women face that are different from MSM. Inclusion of trans women in PrEP campaigns are needed to increase awareness. Changes to CDC guidelines for PrEP that are based on evidence regarding trans women’s HIV risks may positively impact provider knowledge and interest in prescribing PrEP to trans women [47]. Alternatively, PrEP accessibility could be offered to any trans woman who wants it and does not demonstrate medical contraindications. This approach is well justified given high HIV prevalence and persistent HIV incidence in this disproportionately impacted population [2].

New delivery models, like pharmacy‐delivered PrEP could address provider barriers and trans women’s justifiable healthcare avoidance [48, 49]. Pharmacy‐delivered PrEP programs will have to accept public health insurance and facilitate application to PrEP access programs if they are to be inclusive of trans women in San Francisco. PrEP‐only clinics for trans women may mitigate barriers related to anticipated discrimination or discomfort discussing sexual health and drug use with primary care providers. Structural barriers will also need to be addressed. Trans women most at risk of HIV are those facing daily threats to their survival. In order for trans women to prioritize HIV prevention and access it, interventions will need to address trans women’s housing and income needs as well. Finally, inclusion of trans women in HIV prevention safety and efficacy trials from the outset [34] is needed to ensure their equitable access to the next generation of biomedical prevention.

## AUTHORS’ CONTRIBUTIONS

ECW and AL designed the study. CMT conducted the analysis and wrote the results. ML, SS, HFR and SA reviewed all results and contributed to writing the manuscript. All authors reviewed and approved the final version.
